# An engineered diatom acting like a plasma cell secreting human IgG antibodies with high efficiency

**DOI:** 10.1186/1475-2859-11-126

**Published:** 2012-09-13

**Authors:** Franziska Hempel, Uwe G Maier

**Affiliations:** 1 LOEWE Center for Synthetic Microbiology (SYNMIKRO), Hans-Meerwein-Strasse, Marburg, D-35032, Germany; 2 Laboratory for Cell Biology, Philipps-Universität Marburg, Karl-von-Frisch Strasse 8, Marburg, D-35032, Germany

**Keywords:** Diatoms, Expression system, IgG antibody, Protein secretion

## Abstract

**Background:**

Although there are many different expression systems for recombinant production of pharmaceutical proteins, many of these suffer from drawbacks such as yield, cost, complexity of purification, and possible contamination with human pathogens. Microalgae have enormous potential for diverse biotechnological applications and currently attract much attention in the biofuel sector. Still underestimated, though, is the idea of using microalgae as solar-fueled expression system for the production of recombinant proteins.

**Results:**

In this study, we show for the first time that completely assembled and functional human IgG antibodies can not only be expressed to high levels in algal systems, but also secreted very efficiently into the culture medium. We engineered the diatom *Phaeodactylum tricornutum* to synthesize and secrete a human IgG antibody against the Hepatitis B Virus surface protein. As the diatom *P. tricornutum* is not known to naturally secrete many endogenous proteins, the secreted antibodies are already very pure making extensive purification steps redundant and production extremely cost efficient.

**Conclusions:**

Microalgae combine rapid growth rates with all the advantages of eukaryotic expression systems, and offer great potential for solar-powered, low cost production of pharmaceutical proteins.

## Background

Microalgae are of great ecological importance as they represent a major source of global oxygen and contribute critically to carbon fixation [1,2]. But also in biotechnical applications microalgae offer enormous potential and have been used in food and cosmetic industry already for many years now as certain species represent a natural source of omega-3-fatty acids, vitamins, pigments and anti-oxidants. Especially within the last decade microalgae came into focus of fuel industry as a renewable and beneficial source of lipid interesting for biodiesel production [3-5].

Another aspect of algal biotechnology is the idea of using microalgae as expression systems for recombinant proteins [6-9]. No matter if enzymes, hormones, antibodies or biotechnological relevant protein compounds – today there is a great demand for recombinant proteins especially in medical and industrial sectors [10]. Classical expression systems like bacteria, yeast or mammalian cell cultures all depend on external carbon sources emerging as an important cost factor in large-scale expression. Microalgae combine various advantages of classical expression systems as they possess rapid growth rates, are very easy to handle, provide eukaryotic post-translational modifications and are no host to human pathogens. Additionally, microalgae are fueled by photosynthesis and work CO_2_-neutral making them very interesting as low-cost environment friendly protein factories [11-13].

Research in that field focused so far mainly on the green alga *Chlamydomonas reinhardtii* demonstrating that medical relevant proteins like antibodies, hormones and vaccines can be produced very efficiently in the chloroplast of the cells [14-18]. Recent work revealed that other species like the diatom *Phaeodactylum tricornutum* can express foreign proteins with high efficiency also from nuclear promoters having the advantage that even complex eukaryotic proteins can be synthesized, which need post-translational modifications and the assembly of multiple subunits. A fully-assembled and functional human IgG antibody against the Hepatitis B Virus surface protein (HBsAg) was shown to accumulate in *P. tricornutum* to 9% of total soluble protein [19]. Furthermore, the introduction of the bacterial PHB-pathway led to efficient production of the bioplastic poly-3-hydroxybutyrate (PHB) demonstrating that algae might represent an production platform not only for proteins but also other bioproducts [20].

Efficient protein expression is an important issue, but before ending up with the final product time consuming and extensive processing steps such as cell harvesting, cell lysis followed by product purification are usually necessary. Hence, the ideal expression system does not only produce recombinant proteins with high efficiency but also secrets the proteins into the medium making many cost-intense purification steps dispensable. So far research on protein secretion in microalgae is very rare, but in cell wall deficient *Chlamydomonas* strains it was already shown that protein secretion of foreign proteins is basically possible even though efficiency seems to be rather low [21]. In diatoms like *P. tricornutum* polysaccharides are known to be secreted depending on culture conditions and the morphotype [22], however, little is known about protein secretion [23-25].

Here we show for the first time that a microalgal system like the diatom *P. tricornutum* is able to secrete a fully assembled and functional human IgG antibody with high efficiency into the medium. Thus, this study highlights the great potential of these microalgae as novel protein factories secreting complex molecules, which remain functional within the medium for several days.

## Results

### Expression and secretion of a human IgG antibody by the diatom *P. tricornutum*

Based on our previous studies on antibody expression in the ER of *P. tricornutum*[19], we now expressed the human IgG antibody against the Hepatitis B Virus surface protein (CL4mAb) without the ER retention signal (DDEL) at the C-terminus of both antibody chains. Amazingly, this modification led to secretion of the fully assembled antibody across the frustule of the diatom and completely functional antibodies accumulated to very high amounts directly in the media.

**Figure 1:**
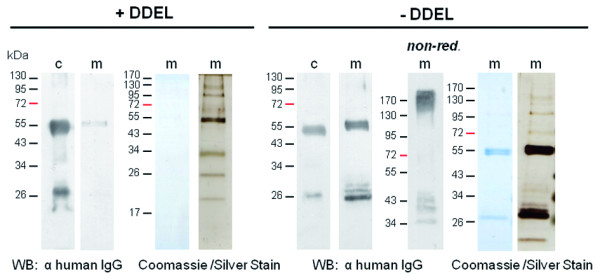
**Analyses on antibody secretion by*P. tricornutum.*** Light and heavy chain of the human IgG antibody CL4mAb against the Hepatitis B Virus surface protein were expressed in *P. tricornutum* for two days either with (+DDEL) or without an ER-retention signal (−DDEL). Proteins of the cellular fraction (c) and the cell-free medium (m) were subsequently separated by gel electrophoresis and analyzed by Western Blot and Coomassie/Silver Staining. In cells expressing an ER-retention signal hardly any antibody is detected in the medium, whereas the deletion of the retention signal leads to efficient antibody secretion. Under non-reduced conditions a high molecular weight signal corresponding to the fully assembled antibody (consisting of two heavy and two light chains) is detected. For Western Blot analyses/Coomassie and Silver Staining proteins of 15 ml/30 ml cell-free medium were concentrated and precipitated. 10 μg protein of the cellular fractions was loaded.

For initial analyses on antibody secretion, expression of recombinant proteins was induced for two days. Subsequently, cells were harvested and the supernatant was filtered to remove any remaining cells. Proteins of the cell-free medium were then concentrated, precipitated and analyzed by gel electrophoresis. Western Blot analyses as well as Coomassie Staining of the gel demonstrated that heavy and light chains are both present in the medium in high quantities and purity as hardly any other proteins were detected even after Silver Staining (Figure 1). In comparison, cells expressing the antibody with an ER-retention signal showed only very low amounts of antibody in the medium (Figure 1). To check in a first approach whether secreted heavy and light chains are assembled in a complex, samples were prepared under non-reduced conditions followed by gel electrophoresis. Western Blot analyses revealed that indeed both chains are no longer detected separately but result in a high molecular weight signal of about 170 kDa (Figure 1), which corresponds to a fully-assembled IgG complex consisting of two heavy and two light chains that are connected via disulfide bridges.

**Figure 2:**
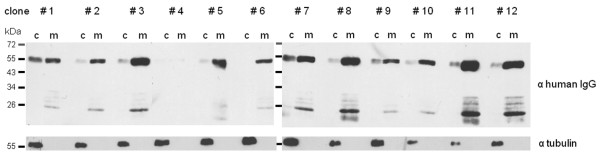
**Comparison of antibody secretion efficiency of different*P. tricornutum*transfectants.** In a small scale screening twelve independent transfectants were tested for antibody expression and secretion efficiency after two days of protein expression. Proteins of the cellular fraction (c) and the cell-free medium (m) were precipitated and separated by SDS-PAGE. Western Blot analyses demonstrate that except for cell line #4 all transfectants express the antibody and secrete it efficiently into the medium. Cell lines #3, #8, #11 and #12 were selected four broader analyses on antibody functionality and quantity. As a loading control for cellular proteins and to check that the medium is not contaminated with intracellular proteins of destroyed cells an antibody against α-tubulin was used. 10 μg protein of the cellular fraction and protein of 15 ml cell-free medium were loaded.

In the following, a set of independent transfectants was tested for antibody secretion to see whether secretion is a rare exception occurring accidentally in some cell lines or whether antibody secretion occurs routinely with differences in secretion efficiency. In a small scale screening twelve independent cell lines were cultivated under non-induced conditions until reaching a density of about 0.2 (OD_600_). Subsequently, cells were transferred to nitrate containing medium for two days and proteins of the cell-free medium were concentrated, precipitated and used for subsequent Western Blot analyses. Except for one cell line, which did not produce detectable antibody levels, all other cell lines tested expressed and secreted the antibody with varying efficiency (Figure 2). Western Blot analyses with an antibody against α-tubulin demonstrate additionally that the medium is free of intracellular proteins, which could have been a result of broken cells. Hence, the detected antibody in the medium is exclusively a result of secretion. In the following, four cell lines with high secretion efficiency (#3, #8, #11 and #12) were selected for broader analyses on functionality and quantification of the secreted antibody.

### Quality and quantity of secreted antibodies

**Figure 3:**
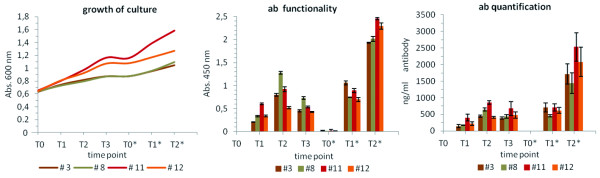
**Analyses on functionality and quantity of secreted antibodies in four independent cell lines.** Antibody expression in cell lines #3, #8, #11 and #12 was induced at time point T0 with a culture density of 0.65. Subsequently, samples of the medium were taken over three days (T1, T2, T3) followed by a medium exchange (T0*) and two additional days of sampling (T1*, T2*). Functionality of the antibody, i.e. the efficiency of the antibody to bind to its antigen, was measured by ELISA assays. The secreted antibody binds to the antigen with an efficiency that basically correlates with the antibody amounts measured in the samples. During the first two days antibody quantities within the medium increased to concentrations of 450–850 ng/ml. After day two the amount of antibody in the medium slightly decreased, which is consequently accompanied by a loss in binding efficiency. The removal of the antibody containing medium and addition of fresh nitrate-containing medium restored antibody expression/secretion and resulted in antibody concentrations of 1550–2550 ng/ml at day T2* depending on the cell line tested. Error bars indicate standard deviation (n = 3).

Western Blot analyses with non-reduced samples of the secreted antibodies gave first indications that indeed fully-assembled antibodies and not only separate antibody chains are secreted by *P. tricornutum* (Figure 1). Most important, however, is of course the question whether i) the secreted antibodies are functional, i.e. are able to recognize the respective target antigen, and ii) the algal expression system can provide secreted antibodies in amounts that are economically interesting. Hence, to check quality and quantity of the secreted antibodies, samples of the medium were taken over a time of five days and analysed by ELISA as well as quantification assays (Figure 3). ELISA analyses demonstrated that the secreted antibodies are indeed functional and recognize the target, the Hepatitis B Virus surface antigen (HBsAg), whereupon binding efficiency basically correlated with the amount of antibodies measured in the medium. The amount of secreted antibodies increased over the first two days of expression (T1,T2) to 450–850 ng antibody per ml medium being reflected in an increased binding efficiency in the ELISA assays as well (Figure 3). After two days the amount of antibody and also binding efficiency slightly decreased, which might be due to degradation in combination with low nitrate levels impairing expression of fresh antibody from the nitrate-inducible promoter, however, by exchanging the medium, and thereby giving a fresh source of nitrate (T0*), antibody expression could be enhanced again and culminated after two days with impressive levels of 1550–2550 ng/ml depending on the cell line tested (Figure 3). Altogether, one can conclude from the data that antibody levels in the medium correlate with the density of the cultures as at later time points T1* and T2* with a culture density of 1.4- 1.6 in cell line #11 antibody amounts are much higher compared to time points T1 and T2. Two days turned out to be an optimal induction period for antibody expression with highest secretion efficiency. The antibody containing medium can then be harvested and replaced with fresh nitrate-medium making a continuous cultivation strategy feasible and very attractive.

## Discussion

Classical expression systems for recombinant proteins like bacteria and yeast have been engineered to produce proteins with high efficiency, however, there are still many drawbacks when expressing complex eukaryotic proteins needing posttranslational modifications or the assembly of multiple protein units. Mammalian systems represent an alternative and are today used for 60–70% of recombinant protein pharmaceuticals, however cultivation is very expensive and always bears the risk of human pathogenic contaminations [26].

Pioneer projects have shown that microalgae perform very well in producing recombinant proteins with the advantage that no external carbon source is needed, which is an important cost factor when large scale production is intended. Microalgae combine rapid growth rates with all advantages of eukaryotic expression systems. Furthermore, many microalgae provide valuable side products interesting for biofuel industry as well as for food and cosmetic sectors [27-30] making microalgae an attractive platform for the production of recombinant proteins.

Monoclonal antibodies belong to one of the largest categories of biotechnologically produced pharmaceuticals today and are needed in diagnostics as well as in therapeutic applications with tumor therapy as a promising novel field of application [31,32]. Many different expression systems were tested over the last 20 years, however, mammalian systems still represent the first choice [33-35]. In previous studies we have shown that a human IgG antibody against the Hepatitis B Virus surface protein can be produced very efficiently in the diatom *P. tricornutum* accumulating to about 9% of total soluble protein. Our data presented here demonstrate additionally that the diatom is able to secrete these antibodies efficiently into the medium. The secreted antibodies are fully-assembled and functional in ELISA and accumulate in the medium to up to 2.5 μg/ml. From an economical point of view the secretion of recombinant proteins into the culture medium is of course an enormous advantage since many processing steps like cell harvesting and lysis are redundant. Because the diatom is not known to secrete many proteins by natural means, the antibodies in the medium are already very pure as confirmed by Coomassie/Silver Staining of precipitated proteins from the medium (Figure 1).

In our studies we have used a nitrate-inducible promoter system, which has the advantage that antibody production is tightly controlled and best induction periods for highest production efficiency with best functionality can be identified. Secretion efficiency in *P. tricornutum* turned out to be highest after 2 days of protein expression with a culture density of 1.0 - 1.6 (OD_600_). The antibodies remained stable within the medium for at least two days before showing a slight decrease in functionality and quantity. Exchanging the culture medium demonstrates that productivity of the cells can be restored when providing fresh nitrate, vitamins etc., hence, a continuous cultivation model seems to be very attractive with the antibody containing medium being harvested and replaced by fresh nitrate-containing medium every two days.

The cell lines used in the presented study have so far been shown to be stable for two years, but when stored probably clones should be stable for many years as known from other *P. tricornutum* transfectants tested in previous studies. First indications suggest additionally that only completely assembled antibodies can get secreted by *P. tricornutum* but no heavy chains or heavy chain dimers (data not shown). Interestingly, this alga seems to have mechanisms similar to mammalian cells to guarantee that heavy chains leave the cell only in association with light chains - “virtually acting like a human plasma cell”. In mammalian cells this is known to be mediated by a stable association with BiP until light chains get bound [36].

## Conclusions

This study highlights the enormous potential of microalgae as solar-fueled expression system for recombinant proteins. Even complex pharmaceutical molecules like completely assembled and functional IgG antibodies can not only be produced in an algal system but also secreted very efficiently into the culture medium. This massively eases downstream purification steps being always problematic and cost-intensive in recombinant protein production. Of course the algal system can presently not compete with mammalian systems that have been engineered to produce high amounts of antibodies with transient expression levels for recombinant antibodies of 100–1000 mg/l [35]. Nevertheless, this pilot projects highlights the great potential of microalgal expression systems, and in future expression of other antibodies as well as production and secretion performance will be optimized. Cells might also be engineered to allow human specific modifications such as specific glycosylation patterns, which was already shown to be feasible in plant systems [37-41] and would broaden the application spectra significantly.

## Methods

### Plasmid construction and *P. tricornutum* transfection

DNA sequences for light and heavy chain of the monoclonal IgG1/kappa antibody CL4mAb were adapted to *P. tricornutum* specific codon usage (GenBank accession numbers: JF970211, JF970210) and cloned into the plasmid pPha-DUAL[2xNR] (JN180664), which contains two multiple cloning sites both under control of the nitrate reductase promoter/terminator system of *P. tricornutum*. Variants with and without an ER retention signal (DDEL) at the C-terminus were generated and introduced into *P. tricornutum* as described previously [19,42].

### Cell culture and analyses on antibody secretion

Cells were cultivated under continuous illumination (80 μmol photons per m^2^ per s) either on plates containing solid f/2-medium with 1.3% agar or in liquid culture with constant agitation (150 rpm) as described previously [19]. For analyses on antibody secretion cells were grown in 50–500 ml f/2 medium with 1.5 mM NH_4_Cl to a density of 0.2-0.7 (OD_600_). Subsequently, cells were harvested (1500 x g, 10 min) and transferred to fresh medium containing 0.9 mM NaNO_3_ to induce antibody production. For initial analyses on antibody secretion and screening for cell lines with highest secretion efficiency cultures were adjusted to the same density (OD_600_ = 0.2) and samples were taken after 2 days of antibody expression. Cells were removed by centrifugation and the medium was filtrated (pore size 0.2 μm) and then concentrated with centrifugal filter columns (cut off 10 kDa). For checking on antibody assembly by SDS-PAGE no β-mercaptoethanol was added to the sample buffer (non-reducing conditions). For broader analyses on production efficiency and antibody functionality over time 1 ml samples were taken after different time points, the medium was filtered and stored at −80°C before proceeding with antibody quantification and functionality analyses.

### Antibody quantification and ELISA

For quantification of the secreted antibody the Easy-Titer Human IgG (H + L) assay kit (ThermoScientific) was used. The filtrated medium was diluted 1:2–1:8 and antibody concentrations were measured according to manufacturer´s instructions with human IgG as standard (550–17.2 ng/ml). Functionality of the secreted antibody was assayed by ELISA. Plates were coated with 200 ng of Hepatitis B Virus surface antigen (HBsAg subtype adr, Abcam) over night as described previously [19]. After blocking and subsequent washing steps, the wells were incubated with the filtrated medium (1:20 dilution in PBS) for 3h. Antibody bound to the HBsAg was detected with an anti-human IgG secondary antibody coupled to HRP (Sigma-Aldrich). Medium of wild type cells and non-induced cultures served as a negative control in both assays. All measurements were carried out in triple.

## Abbreviations

HBsAg, Hepatitis B Virus surface antigen; IgG, Immunoglobulin G; PHB, Poly-3-hydroxybutyrate.

## Competing interests

The Philipps-Universität Marburg and the authors of the manuscript applied for a patent relating to the content of the manuscript (EP 12170995.0).

## Authors’ contributions

FH designed and conducted the experiments and together with UGM analyzed the data and wrote the manuscript. All authors read and approved the final manuscript.
